# Supplementary material for: Cell-lysis sensing drives biofilm formation in Vibrio cholerae
Source: Nat Commun. 2024 Mar 6;15:2018. doi: 10.1038/s41467-024-46399-1 (PMC10914755; doi:10.1038/s41467-024-46399-1)
Supplement: Supplementary file 1 — Supplementary Information [file 41467_2024_46399_MOESM1_ESM.pdf]

Supplementary Information for

Cell-lysis sensing drives biofilm formation in *Vibrio cholerae*

Jojo A. Prentice, Robert van de Weerd, and Andrew A. Bridges*

Corresponding author: bridges@cmu.edu

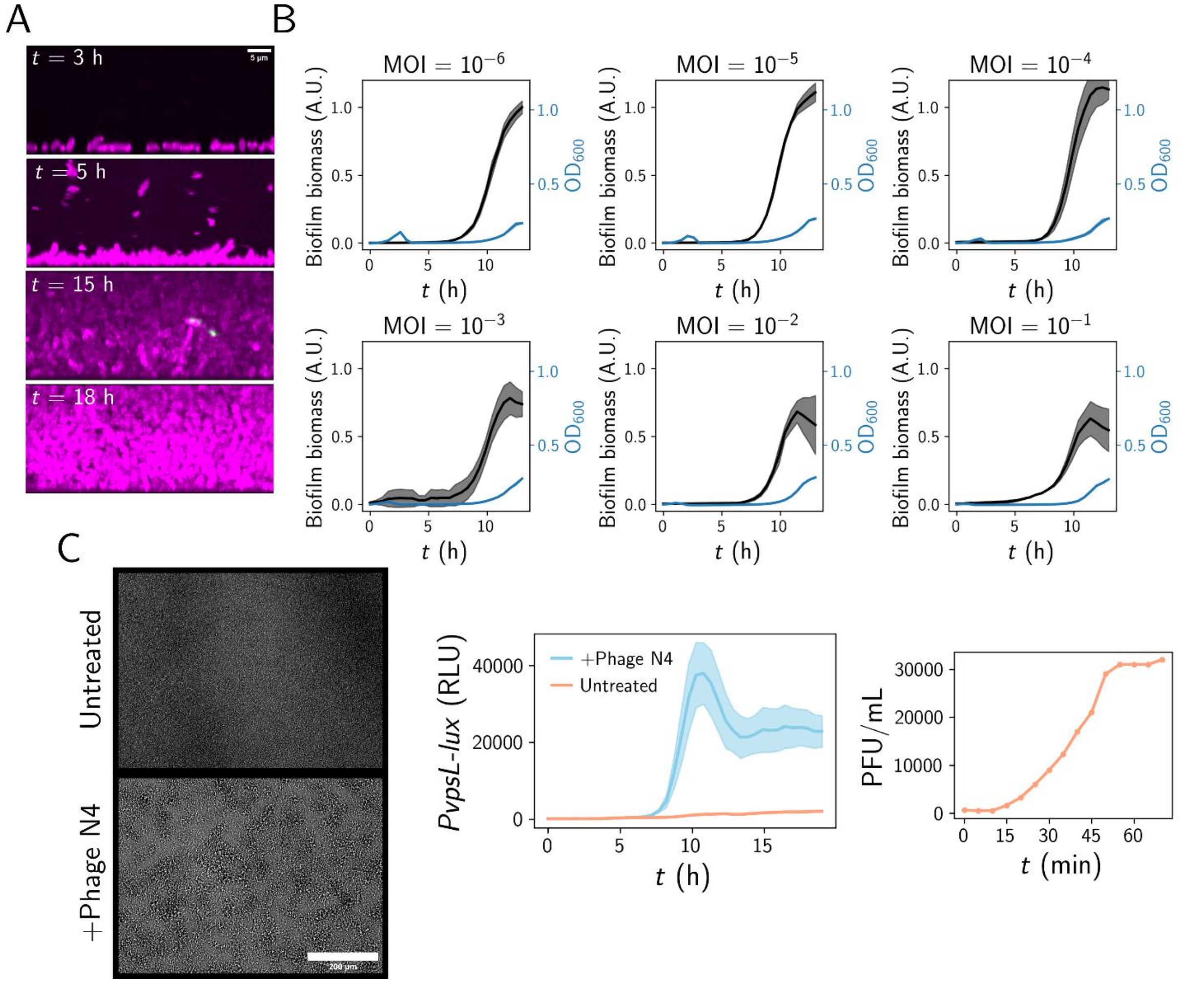

Supplementary Fig. 1. Characterization of the phage-biofilm response in *V. cholerae* (A) Side-on (XZ) projections of confocal images for the indicated timepoints of *V. cholerae* grown without phage, in which the field of view becomes filled with planktonic cells over time. Magenta represents live cells expressing a constitutive reporter and green represents dead cells stained with SYTOX. Scale bar is indicated in the top panel. (B) OD_{600} growth curve and biofilm biomass curve as measured by brightfield microscopy for wildtype *V. cholerae* grown in the presence of phage S5 at the indicated MOI's. Phage S5 was prepared via filtration through a 0.22 µm filter. (C) Left: representative brightfield images of wildtype *V. cholerae* cultures grown in the absence (top) or presence (bottom) of phage N4 (MOI = 10^{-6}). Displayed images are of cultures at equivalent cell densities. Scale is as indicated in the bottom panel (200 µm). Middle: *PvpsL-lux* output over time from wildtype *V. cholerae* grown in the indicated conditions. The phage treated cultures were MOI = 10^{-6}. Right: one-step growth curve for phage N4 at 32°C. A. U.; arbitrary units. In B and C (middle panel), data are averages of n = 3 biological replicates, and shading represents standard deviations. A.U.; arbitrary units. RLU; relative-light units.

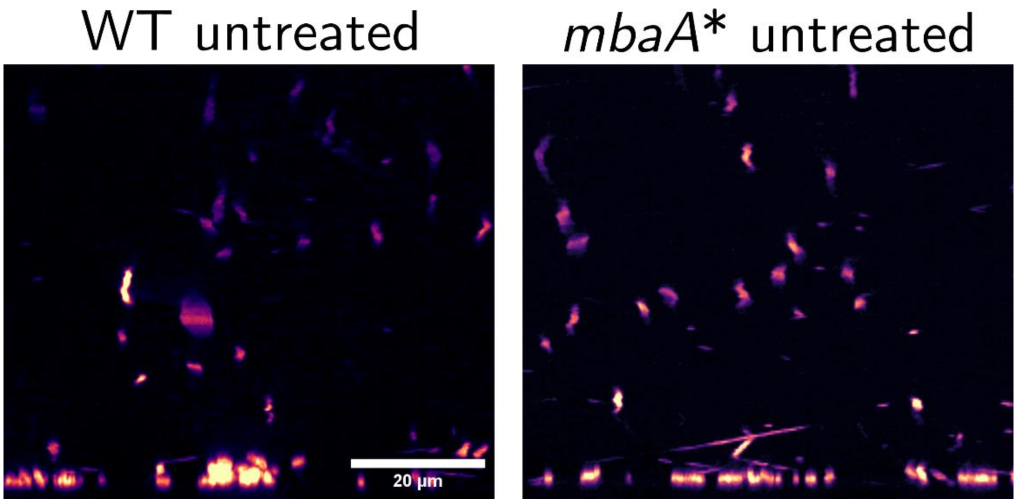

Supplementary Fig. 2. Representative images of the WT and *mbaA strains without lysate.** Images represent XZ confocal projections of the indicated strains after 16 hours of growth in the absence of lysate. Cells were stained with MM4-64, displayed with the mpl-magma look-up table. Scale is as indicated in the left panel.

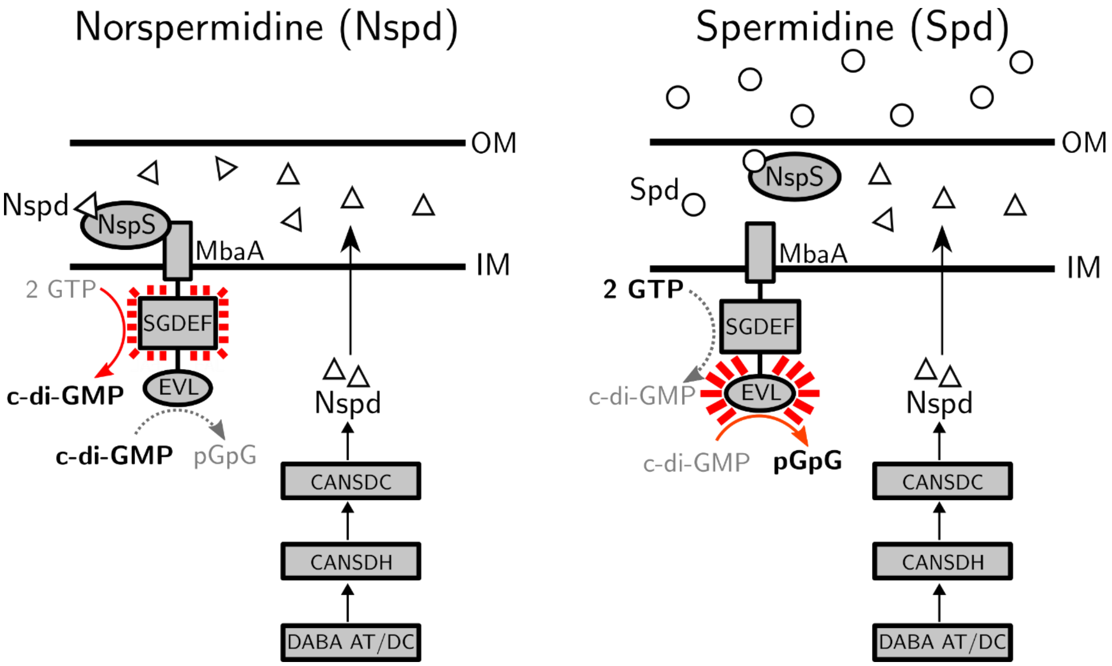

Supplementary Fig. 3. Schematic of the norspermidine/spermidine signaling pathway in *V. cholerae*. Left: signaling response to norspermidine (Nspd). When norspermidine is abundant in the periplasm of *V. cholerae*, it binds the periplasmic protein NspS, and the Nspd-NspS complex in turn associates with the transmembrane receptor MbaA. MbaA is a bifunctional receptor capable of both synthesizing and degrading the intracellular second messenger c-di-GMP. When bound by the Nspd-NspS complex, MbaA is biased toward c-di-GMP synthesis and as a result, c-di-GMP levels become elevated in the cytoplasm driving biofilm gene expression. The *mbaA** mutant we used in our experiments encodes an MbaA protein deficient in c-di-GMP synthesis activity, and therefore, the strain does not respond to Nspd. Right: signaling response to spermidine (Spd). Spermidine is produced by species outside the *Vibrio* genus, and so *V. cholerae* cells only encounter it from an external source, as indicated. When spermidine is abundant in the periplasm and norspermidine is not, spermidine binds NspS, displacing it from its MbaA binding site and biasing MbaA toward c-di-GMP degradation. Consequently, c-di-GMP concentrations decrease in the cytoplasm and the cell inactivates biofilm gene expression. Norspermidine is synthesized in the *V. cholerae* cytoplasm via a biosynthetic pathway whose first catalytic step is carried out by a unique enzyme DABA AT/DC, which is a fusion of the common L-2,4-diaminobutyrate aminotransferase (DABA AT) and L-2,4-diaminobutyrate decarboxylase (DABA DC) enzymes. This enzyme is thought to be required for norspermidine synthesis. The product of DABA AT/DC is subsequently processed by the enzymes carboxynorspermidine dehydrogenase (CANSDH) and carboxynorspermidine decarboxylase (CANSDC, the gene product of *nspC*) to yield norspermidine, which can then be exported to the periplasm or remain in the cytoplasm for other functions.

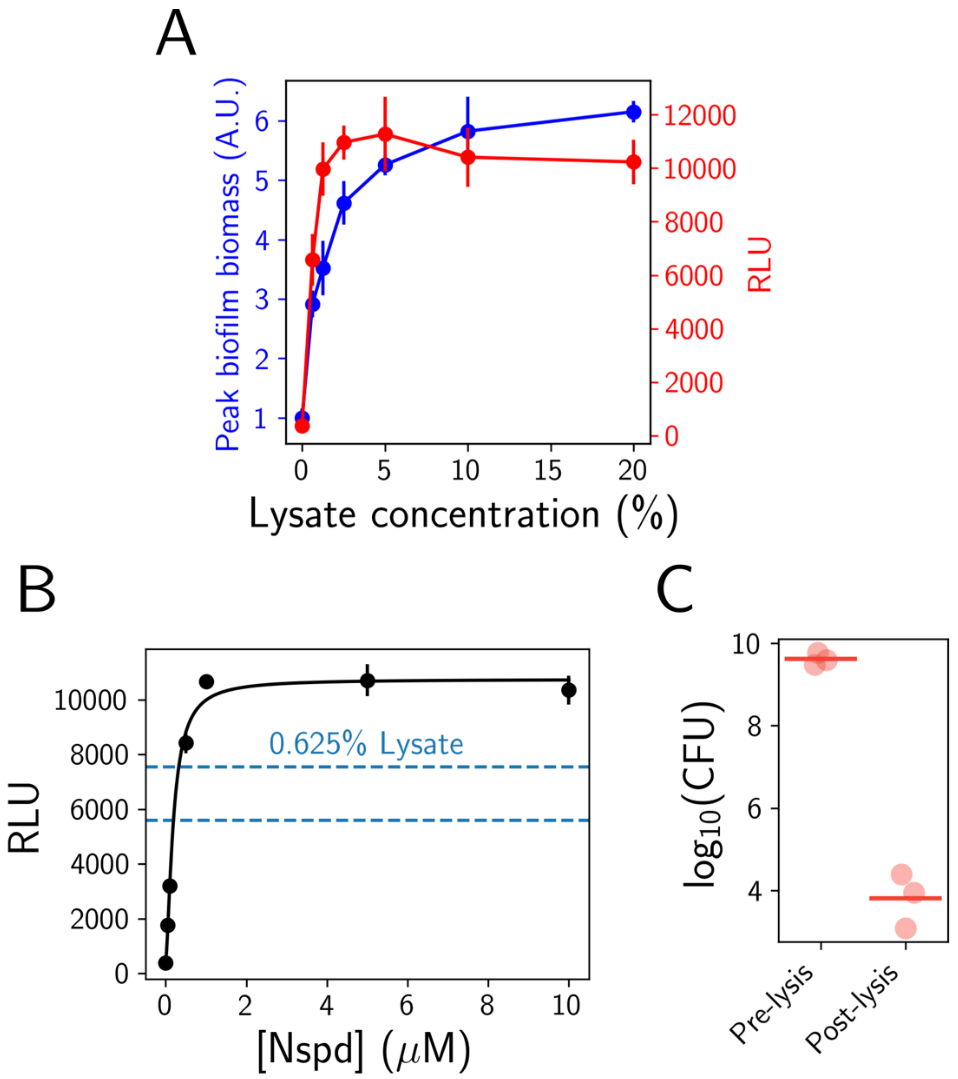

Supplementary Fig. 4. Quantitative characterization of the norspermidine concentration in *V. cholerae* lysate and the dose-response lysate-biofilm relationship. (A) Peak biofilm biomass (blue) and peak P*vpsL-lux* output (red) of wildtype *V. cholerae* in the presence of the indicated lysate concentrations. Points represent averages and error bars represent standard deviations. Biofilm biomass data are normalized as fold-change relative to the untreated condition. (B) Peak P*vpsL-lux* output of wildtype *V. cholerae* in the presence of 20% Δ*nspC* lysate and the indicated norspermidine concentrations (black dots). Points represent averages and error bars represent standard deviations. Blue dotted lines represent the lower and upper bounds (by standard deviation) of the peak P*vpsL-lux* output of wildtype *V. cholerae* treated with 0.625% wildtype lysate. Black curve represents a hill function fitted to the dose-response data (R^2 = 0.994). (C) Recovery (CFUs) of a wildtype *V. cholerae* overnight culture before and after ten freeze-thaw cycles. In all cases, data represent *n*=3 biological replicates. RLU; relative-light units. CFU; colony-forming units. Nspd; norspermidine. A.U.; arbitrary units.

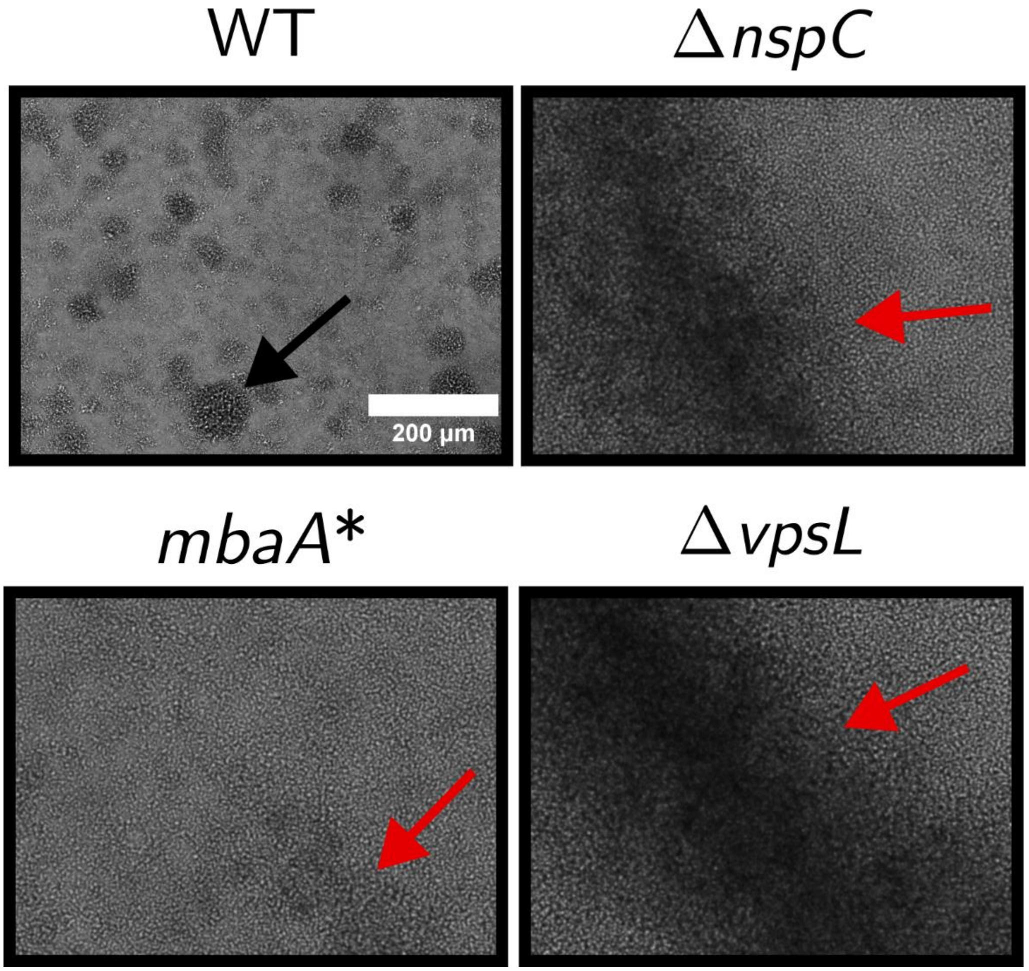

Supplementary Fig. 5. Representative brightfield images of wildtype, Δ*nspC*, *mbaA, and Δ*vpsL* mutants grown in the presence of phage S5.** Images are from the 15-hour timepoint of growth with phage S5. The black arrow in the WT image indicates VPS-dependent biofilms, which are segmented in our image analysis pipeline. Red arrows indicate more diffuse aggregate-like biofilms (red) which form in the mutants and do not meet the threshold conditions in our image analysis pipeline. Scale is as indicated in the top left image.

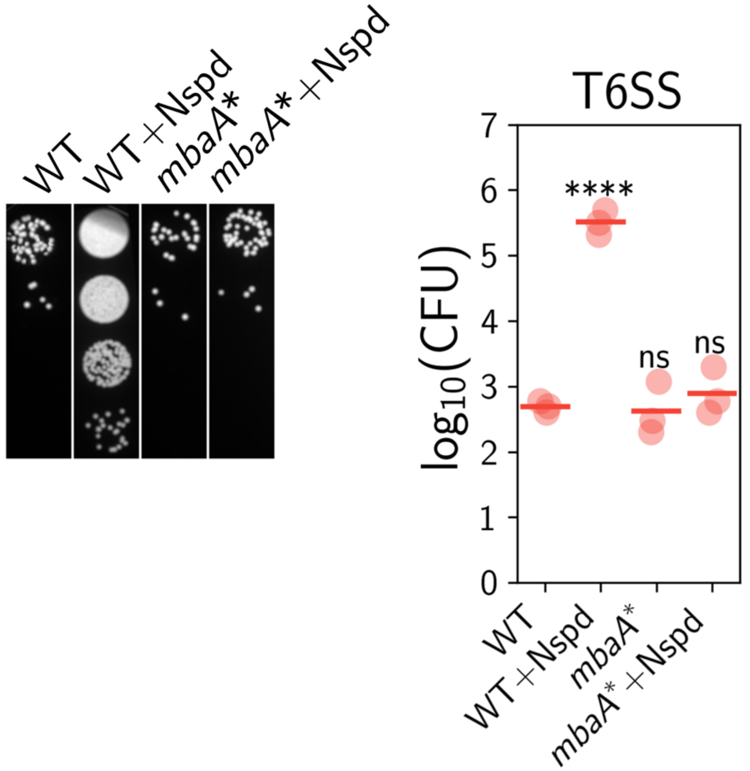

Supplementary Fig. 6. The norspermidine-induced biofilm state protects *V. cholerae* cells against Type-VI secretion system attacks. Log_{10} CFU of the indicated *V. cholerae* strains and treatment conditions after 2 hours of exposure to *A. baylyi* ADP1. Data represent *n=3* biological replicates. Points represent individual replicates and crossbars represent means. Unpaired, two-sided *t*-tests relative to the control (WT) were performed for statistical significance. $p = 1.96 \times 10^{-5}$, 7.74×10^{-1}, and 4.05×10^{-1} for the indicated comparisons. A.U.; arbitrary units. CFU; colony-forming units. Nspd; norspermidine. ****; $p \leq 0.0001$. ns; $p > 0.05$.

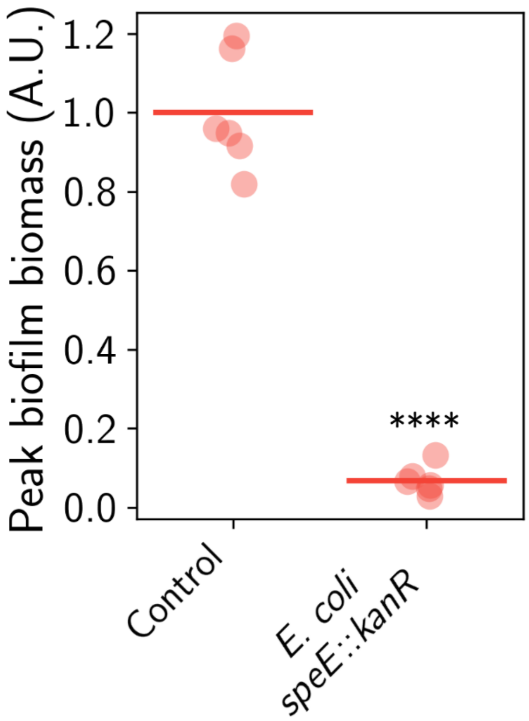

Supplementary Fig. 7. Repression of biofilm formation by lysates produced from mechanical disruption is not due to the presence of spermidine. Peak biofilm biomass of *V. cholerae* in the absence of lysate and in the presence of lysate produced from *E. coli speE*::*kanR*. Data represent *n*=6 biological replicates of the recipient strain and are normalized to the mean peak biofilm biomass of the control (no lysate). Points represent individual replicates and crossbars represent means. An unpaired, two-sided *t*-test was performed for statistical significance. $p = 3.43 \times 10^{-8}$. A. U.; arbitrary units. ****; $p \leq 0.0001$.

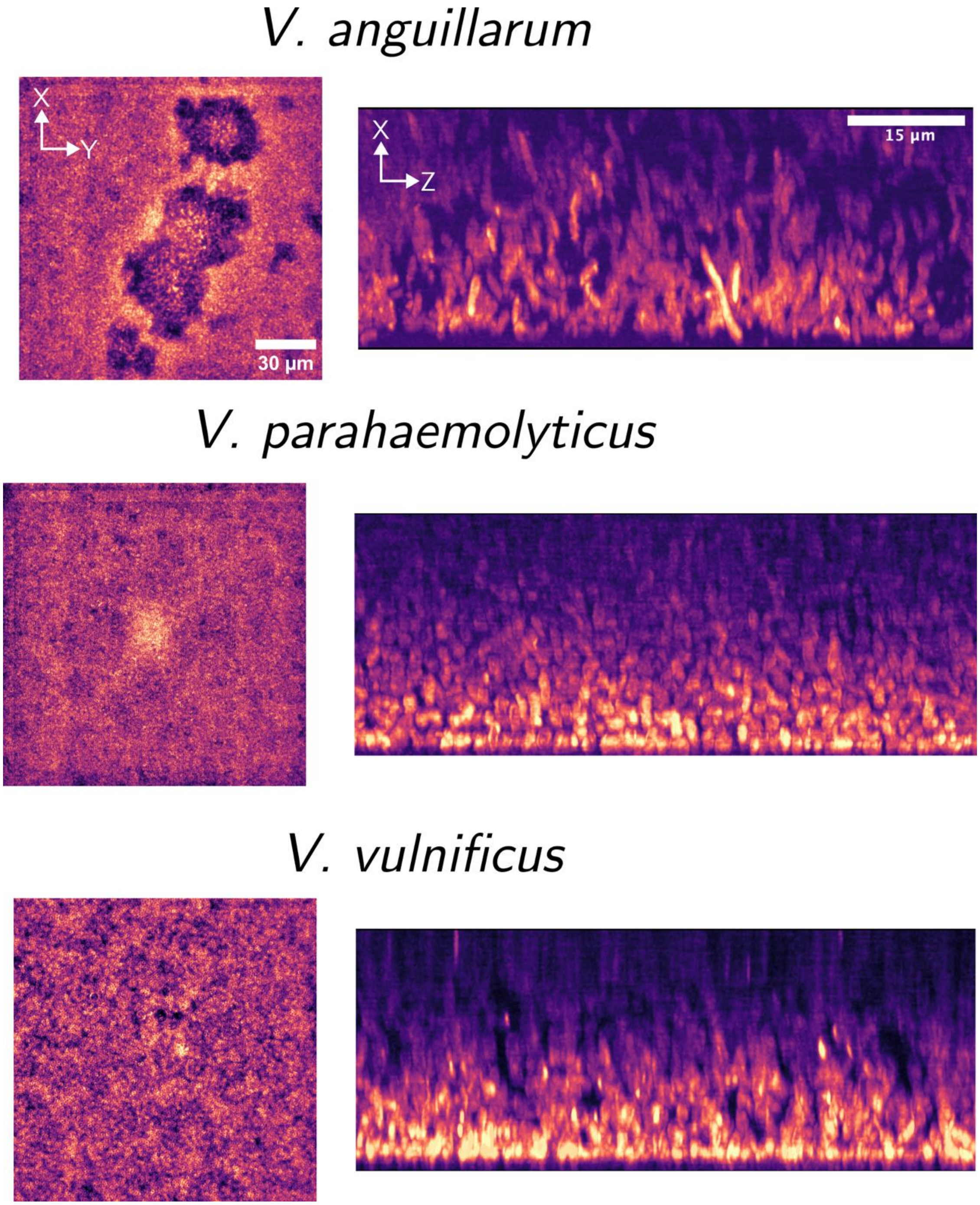

Supplementary Fig. 8. Control images of *V. anguillarum*, *V. parahaemolyticus*, and *V. vulnificus* without lysate. Images represent XY and XZ confocal projections of the indicated strains after 16 hours (*V. anguillarum* and *V. vulnificus*) or 12 hours (*V. parahaemolyticus*). In the images, *V. anguillarum* displays a modest level of biofilm formation, whereas *V. parahaemolyticus* and *V. vulnificus* exist in the planktonic states uniformly across the fields of view. Cells were stained with MM4-64, displayed with the mpl-magma look-up table. Scale and axes are as indicated in the top images.

Supplementary Table 1. Homologs of Vc_1625 (DABA AT/DC), enzyme for norspermidine biosynthesis.

Species*	Sequence identity (%)	Accession length (amino acids)	Accession
Aliivibrio logei	75	967	WP_023603545.1
Aliivibrio salmonicida	75	967	WP_012550664.1
Aliivibrio sifiae	69	968	WP_105062906.1
Aliivibrio wodanis	71	962	CED71812.1
Candidatus Enterovibrio	65	967	WP_097356396.1
Dasania marina	78	960	WP_019528168.1
Enterovibrio baiacu	68	966	WP_129493416.1
Enterovibrio calviensis	68	967	WP_028022701.1
Enterovibrio coralii	68	967	WP_067414160.1
Enterovibrio nigricans	67	967	WP_078754254.1
Enterovibrio norvegicus	68	971	WP_039735468.1
Enterovibrio pacificus	67	973	WP_068904803.1
Enterovibrio paralichthyis	68	967	WP_218357753.1
Grimontia celer	68	967	WP_062660920.1
Grimontia hollisae	66	967	WP_158161014.1
Grimontia indica	69	966	WP_002539286.1
Grimontia marina	68	966	WP_062711180.1
Grimontia sedimenti	68	966	WP_165012932.1
Idotea baltica	79	945	MCL4110690.1
Paraphotobacterium marinum	58	959	WP_089072553.1
Photobacterium alginatilyticum	70	962	WP_160648311.1
Photobacterium angustum	69	969	WP_045153557.1
Photobacterium aphoticum	72	966	GAL09018.1
Photobacterium aquae	71	968	KLV07031.1
Photobacterium arenosum	69	961	WP_192016477.1
Photobacterium atrarenae	69	962	WP_255390129.1
Photobacterium chitinilyticum	71	962	WP_128782917.1
Photobacterium frigidiphilum	70	961	WP_107242154.1
Photobacterium gaetbulicola	71	965	WP_039468551.1
Photobacterium galatheae	70	961	WP_036750685.1
Photobacterium ganghwense	71	967	WP_047885699.1
Photobacterium halotolerans	70	958	KKD01207.1
Photobacterium indicum	71	961	WP_107253878.1
Photobacterium jeanii	71	967	WP_068332556.1
Photobacterium leiognathi	70	968	WP_045065155.1
Photobacterium lipolyticum	70	962	WP_107281362.1
Photobacterium lucens	69	968	WP_161118161.1
Photobacterium lutimaris	70	965	WP_107348356.1
Photobacterium marinum	71	962	WP_007462339.1
Photobacterium profundum	71	961	WP_086004185.1
Photobacterium proteolyticum	70	962	WP_075766363.1
Photobacterium rosenbergii	71	965	WP_107297577.1
Photobacterium salinisoli	69	961	WP_120510614.1
Photobacterium sanctipauli	71	965	WP_107271636.1
Photobacterium sanguinicancri	71	965	WP_062690892.1

Photobacterium swingsii	71	966	KMV28663.1
Vibrio aerogenes	77	957	WP_073605928.1
Vibrio aestuarianus	87	961	WP_172532192.1
Vibrio agarilyticus	73	960	WP_168837129.1
Vibrio agarivorans	79	966	WP_264877368.1
Vibrio alfacsensis	83	963	WP_221069204.1
Vibrio alginolyticus	84	958	MCA0936142.1
Vibrio algivorus	75	973	WP_144387339.1
Vibrio amylolyticus	78	962	WP_248010158.1
Vibrio anguillarum	86	962	WP_194662732.1
Vibrio antiquarius	84	958	MCR9986648.1
Vibrio aphrogenes	75	973	WP_086984116.1
Vibrio aquaticus	82	971	WP_126573767.1
Vibrio aquimaris	79	965	WP_152430545.1
Vibrio artabrorum	79	983	WP_261837528.1
Vibrio astriarenae	79	966	WP_164648313.1
Vibrio atlanticus	79	975	TVU59427.1
Vibrio atypicus	82	968	WP_159739016.1
Vibrio azureus	80	958	WP_021709101.1
Vibrio barjaei	77	965	WP_268678706.1
Vibrio bathopelagicus	79	983	WP_192888956.1
Vibrio bivalvicida	81	966	WP_054963003.1
Vibrio brasiliensis	81	966	WP_006880993.1
Vibrio breoganii	76	963	WP_102477458.1
Vibrio campbellii	84	958	WP_077200511.1
Vibrio caribbeanicus	84	963	WP_038134452.1
Vibrio casei	75	976	WP_193013883.1
Vibrio celticus	79	983	WP_170926835.1
Vibrio chagasii	79	979	WP_239930979.1
Vibrio chemaguriensis	83	958	WP_193447737.1
Vibrio cholerae	100	961	WP_000100702.1
Vibrio cidicii	87	959	WP_061899759.1
Vibrio cincinnatiensis	91	961	WP_238132389.1
Vibrio comitans	76	963	WP_141271478.1
Vibrio coralliilyticus	84	965	WP_171347724.1
Vibrio coralliirubri	79	983	WP_181949954.1
Vibrio cortegadensis	79	965	WP_261888320.1
Vibrio crassostreae	79	983	WP_017061003.1
Vibrio cyclitrophicus	80	911	WP_102270524.1
Vibrio diabolicus	84	958	CDT89733.1
Vibrio diazotrophicus	92	961	WP_102953067.1
Vibrio echinoideorum	79	983	WP_210114703.1
Vibrio eleionomae	82	962	WP_161157275.1
Vibrio europaeus	82	966	WP_272273649.1
Vibrio ezurae	75	963	WP_021713157.1
Vibrio fluvialis	92	961	MBY7789126.1
Vibrio fortis	79	973	WP_261903930.1
Vibrio furnissii	92	961	WP_004725626.1
Vibrio galatheae	85	966	WP_045957101.1

Vibrio gallaecicus	79	971	WP_137372558.1
Vibrio gallicus	75	965	WP_261817252.1
Vibrio gangliei	75	973	WP_105901965.1
Vibrio gazogenes	80	962	WP_072957394.1
Vibrio gelatinilyticus	77	965	WP_244354179.1
Vibrio genomosp.	78	965	WP_017054571.1
Vibrio gigantis	79	983	WP_239716186.1
Vibrio halioticoli	75	963	WP_023404221.1
Vibrio hangzhouensis	78	965	WP_103881936.1
Vibrio harveyi	84	963	EEZ85480.1
Vibrio hepatarius	85	962	WP_171384442.1
Vibrio hibernica	75	973	WP_194089372.1
Vibrio hippocampi	77	962	WP_237484685.1
Vibrio hyugaensis	84	958	WP_045460219.1
Vibrio ichthyoenteri	78	964	WP_006710950.1
Vibrio injensis	91	961	WP_072669445.1
Vibrio inusitatus	76	963	WP_141345357.1
Vibrio ishigakensis	75	965	WP_261835430.1
Vibrio japonicus	85	962	WP_257085401.1
Vibrio jasicida	84	963	WP_038876061.1
Vibrio kanaloae	79	983	WP_208806546.1
Vibrio lentus	80	975	PML46238.1
Vibrio litoralis	76	972	WP_027696056.1
Vibrio maerlii	78	964	WP_117234606.1
Vibrio mangrovi	81	962	WP_087480085.1
Vibrio marinisediminis	78	961	WP_182109613.1
Vibrio marisflavi	80	960	WP_237362237.1
Vibrio maritimus	78	965	WP_234494039.1
Vibrio mediterranei	78	965	WP_088875869.1
Vibrio metoecus	99	961	WP_154171740.1
Vibrio metschnikovii	91	961	WP_217515595.1
Vibrio mexicanus	80	969	WP_047042542.1
Vibrio mimicus	97	961	WP_000100710.1
Vibrio mytili	84	958	WP_041155457.1
Vibrio natriegens	85	958	WP_065302587.1
Vibrio navarrensis	86	959	WP_193152658.1
Vibrio neocaledonicus	83	958	QCO86508.1
Vibrio neonatus	75	963	WP_261824105.1
Vibrio neptunius	83	965	WP_045973859.1
Vibrio nereis	85	962	WP_282176523.1
Vibrio nigripulchritudo	81	962	WP_200767060.1
Vibrio nitrifigilis	82	962	WP_196123252.1
Vibrio ordalii	86	962	OEE76606.1
Vibrio orientalis	80	972	WP_004414227.1
Vibrio ostreae	91	961	WP_218563038.1
Vibrio ostreicida	80	962	WP_076587644.1
Vibrio ouci	81	972	WP_134834874.1
Vibrio owensii	84	958	WP_199454828.1
Vibrio pacinii	84	961	WP_038174602.1

Vibrio panuliri	76	964	WP_075707539.1
Vibrio paracholerae	100	961	WP_252921195.1
Vibrio parahaemolyticus	85	770	OTW26533.1
Vibrio paucivorans	79	965	WP_265688119.1
Vibrio pectenicida	78	967	WP_125320812.1
Vibrio pelagius	78	973	WP_255230926.1
Vibrio penaeicida	81	962	WP_126606689.1
Vibrio plantisponsor	91	961	WP_171137633.1
Vibrio pomeroyi	78	983	WP_261884583.1
Vibrio ponticus	78	961	GAK84550.1
Vibrio porteresiae	83	962	WP_261895908.1
Vibrio proteolyticus	81	960	WP_021705446.1
Vibrio qingdaonensis	75	967	WP_265673491.1
Vibrio qinghaiensis	86	962	WP_094500016.1
Vibrio quintilis	77	957	WP_073582345.1
Vibrio rarus	75	963	WP_261874545.1
Vibrio rhizosphaerae	80	962	WP_038178452.1
Vibrio rhodolitus	77	961	WP_114767134.1
Vibrio rotiferianus	84	958	WP_045387787.1
Vibrio ruber	80	962	WP_077335796.1
Vibrio rumoiensis	75	972	WP_089138721.1
Vibrio sagamiensis	78	958	WP_039980509.1
Vibrio salilacus	84	961	WP_100752245.1
Vibrio scophthalmi	78	970	WP_069446731.1
Vibrio sinaloensis	84	963	WP_038192530.1
Vibrio sinus	79	959	WP_250612660.1
Vibrio sonorensis	80	962	WP_070967680.1
Vibrio spartinae	79	962	WP_182288541.1
Vibrio splendidus	80	975	PMK15701.1
Vibrio stylophorae	67	963	WP_237465389.1
Vibrio superstes	76	963	WP_119010341.1
Vibrio tapetis	78	962	WP_102521253.1
Vibrio tarriae	100	961	WP_113598746.1
Vibrio tasmaniensis	79	975	PMP10618.1
Vibrio tetraodonis	78	965	WP_160932905.1
Vibrio thalassae	77	965	WP_096994338.1
Vibrio toranzoniae	78	975	WP_161678138.1
Vibrio tritonius	83	962	WP_068713472.1
Vibrio tubiashii	84	963	KGY12235.1
Vibrio ulleungensis	78	963	WP_205159887.1
Vibrio variabilis	84	963	WP_038212014.1
Vibrio viridaestus	80	960	WP_124936920.1
Vibrio vulnificus	86	959	HAS6233080.1
Vibrio xiamenensis	79	960	WP_093271101.1
Vibrio xuii	81	972	KOO16586.1
Vibrio zhanjiangensis	78	965	GLT16812.1
Vibrio ziniensis	91	961	WP_165311564.1

*Species used in this study are bolded.

Supplementary Table 2. Homologs of Vc_0703 (MbaA), receptor for norspermidine.

Species*	Sequence identity (%)	Accession length (amino acids)	Accession
Vibrio aestuarianus	67	783	WP_274684396.1
Vibrio alginolyticus	67	783	KOE81085.1
Vibrio anguillarum	66	783	WP_208874291.1
Vibrio cholerae	100	791	WP_000773112.1
Vibrio cortegadensis	62	771	WP_261888518.1
Vibrio diazotrophicus	65	781	WP_042484232.1
Vibrio eleionomae	64	785	WP_161157096.1
Vibrio fluvialis	74	782	MBY8035852.1
Vibrio furnissii	75	782	QTG89158.1
Vibrio gazogenes	65	774	SHF73897.1
Vibrio genomosp.	62	771	WP_017053101.1
Vibrio mangrovi	67	781	WP_087481417.1
Vibrio metoecus	91	791	WP_055043604.1
Vibrio mimicus	92	791	WP_000773100.1
Vibrio nitrifigilis	64	785	WP_196122395.1
Vibrio ordalii	65	783	OEE40523.1
Vibrio ostreae	68	781	WP_218562746.1
Vibrio paracholerae	99	791	WP_206500748.1
Vibrio plantisponsor	64	781	WP_171138191.1
Vibrio porteresiae	68	785	WP_261893491.1
Vibrio proteolyticus	60	785	WP_021705606.1
Vibrio qinghaiensis	65	784	WP_094499727.1
Vibrio ruber	67	774	WP_077333135.1
Vibrio salinus	67	771	WP_232765212.1
Vibrio spartinae	65	774	QMV13469.1
Vibrio tarriae	100	791	WP_113602367.1
Vibrio tritonius	66	785	WP_068714812.1
Vibrio viridaestus	67	772	WP_124937480.1
Vibrio ziniensis	64	781	WP_165310425.1

*Species used in this study are bolded.

Supplementary Table 3. Strains used in this study.

Strain number	Species/Genotype	Plasmid	Antibiotic resistance	Origin
AB_Vc_707	*Vibrio cholerae* C6706	-	-	Bassler lab
AB_Vc_323	Δ*vpsL*	-	-	Jemielita et al., 2018
AB_Vc_823	Δ*nspC* Δ*vc1807*::*kan*^{R}	-	Kan	NT of AB_Vc_707
AB_Vc_870	*mbaA*$^{D426A, E427A}$ Δ*vc1807*::*kan*^{R}	-	Kan	NT of AB_Vc_707
AB_Vc_683	Δ*vc1807*::*Ptac-mScarlet*::*spec*^{R}	-	Spec	NT of AB_Vc_102
JP_Vc_1515	*mbaA*$^{D426A, E427A}$ Δ*vc1807*::*Ptac-mScarlet*::*spec*^{R}	-	Spec	NT of AB_Vc_870
JP_Vc_1517	Δ*nspC* Δ*vc1807*::*Ptac-mScarlet*::*spec*^{R}	-	Spec	NT of AB_Vc_823
AB_Vc_801	*Vibrio cholerae* C6706	pEVS143-*PvpsL-lux*::*CmR*	Cm	Conj of AB_Vc_479
AB_Vc_823	Δ*nspC* Δ*vc1807*::*kan*^{R}	pEVS143-*PvpsL-lux*::*CmR*	Cm	Conj of AB_Vc_823
JP_Vc_1979	*mbaA*$^{D426A, E427A}$ Δ*vc1807*::*kan*^{R}	pEVS143-*PvpsL-lux*::*CmR*	Cm	Conj of AB_Vc_870
RW_Vc_1892	Δ*vc1807*::*Ptac-ssMBP-dl5*::*spec*^{R}	-	Spec	NT of AB_Vc_707
JP_Vc_1959	Δ*vpsL* Δ*vc1807*::*Ptac-ssMBP-dl5*::*spec*^{R}	-	-	NT of AB_Vc_323
JP_Vc_1960	Δ*nspC* Δ*vc1807*::*Ptac-ssMBP-dl5*::*spec*^{R}	-	-	NT of AB_Vc_823
JP_Vc_1962	*mbaA*$^{D426A, E427A}$ Δ*vc1807*::*Ptac-ssMBP-dl5*::*spec*^{R}	-	-	NT of AB_Vc_870
AB_OV_001	*Vibrio campbellii* BB120	-	-	Bassler lab
AB_OV_003	*Aliivibrio fischeri* ES114	-	-	Bassler lab
AB_OV_005	*Vibrio anguillarum* 19264	-	-	Bassler lab
AB_OV_006	*Vibrio natriegens* 77	-	-	Bassler lab
AB_OV_010	*Vibrio parahaemolyticus* RIMD	-	-	Bassler lab
AB_OV_011	*Vibrio vulnificus* JJ077	-	-	Bassler lab
AB_NV_005	*Pseudomonas aeruginosa* PA14	-	-	Hiller lab
IMB_Ec_099	*Escherichia coli* BW25113	-	-	Silhavy lab
IMB_Ec_100	*Escherichia coli speE*::*kan*^{R}	-	Kan	Silhavy lab
AB _NV_009	*Acinetobacter baylyi* ADP1	-	-	Ellison lab
AB_Nv_025	*Vibrio cholerae* phage S-5	-	-	ATCC 51352-B2
JP_Nv_036	*Vibrio cholerae* phage N-4	-	-	ATCC 51352-B1

NT; natural transformation. Conj; conjugation.

Supplementary Table 4. Oligos used in this study.

Oligo number	Name	Purpose		Direction	5' to 3' Sequence
386	Vc1807_1500_up	Cloning constructs at Vc_1807 locus		F	GCTCTAACCGTATATACAACTCCAAGTGG
387	Vc1807_1500_down	Cloning constructs at Vc_1807 locus		R	CCGTTTCATGCCCTACTCGCTAAC
1402	Vc1807_ssMBP_dL5	Gblock for Constitutive dL5 reporter		F	CCTAGGAATTCAATTAGGAGGTAATTAAGCTTGGTGAGCCCTACAACACAAAAAGAAAAGGATATGAACATGAAAAATGCCCTAAGCACAGTCGCGCTGAGCACTCTGGTGGCTCTTGGTTCGTTTGGTGCCCATGCTGCGAAGCGGAAGCATCTGCTGGCGCTGGGGGCGGAGGCAGCGCGTCTATGCAGGCCGTAGTGACCCAAGAACCCAGTGTGACTGTCAGTCCAGGAGGCACCGTGATCTTAACTTGTGGTTCAGGTACTGGGGCGGTTACCAGCGGTCATTATGCTAATTGGTTTCAGCAAAAGCCAGGACAAGCTCCGCGCGCGCTTATTTTCGACACCGACAAGAAATACTCTTGGACTCCTGGGCGCTTTTCAGGCAGTTTACTGGGCGCGAAAGCGGCTTTGACGATCTCTGACGCACAGCCAGAGGATGAAGCGGAATATTACTGTAGCCTGTCTGACGTAGATGGCTATTTATTTGGCGGAGGTACTCAACTTACGGTGTTGTCCGGAGGCGGTGGGTCGGGCGGTGGGGGCTCCGGAGGTGGCGGAAGCGGAGGGGGAGGCAGCCAAGCAGTGGTTACTCAGGAGCCGTCTGTTACCGTGTCACCGGGCGGTACTGTGATTCTGACCTGCGGCTCGGGAACTGGGGCGGTCACCTCAGGGCACTATGCAAATTGGTTTCAACAGAAACCCGGTCAAGCGCCTCGCGCACTTATCTTTGATACTGATAAGAAATATAGTTGGACGCCAGGTCGTTTCAGCGGTTCTCTGTTGGGCGCAAAAGCTGCGCTTACCATTAGCGATGCGCAACCTGAGGACGAGGCCGAGTACTATTGTTCGCTGTCGGATGTAGATGGATACCTATTTGGTGGCGGAACACAACTAACGGTGCTGTC

					CACCGGGCATCACCATCACCATCACTAAGTGTGAATTCCGGGGATCCGTCGACCTGCAGTTCGA
1390	Rv_Mbp_DL5ovl_5	Cloning Constitutive dL5 reporter		R	TCCGCCCCCAGCGCCAGCAGACAAGCCCCCACCGCCTTTCGTCATCTGCTTTTCAGCATC
1391	Fw_ovlMBP_DL5_6	Cloning Constitutive dL5 reporter		F	GCTTGTCTGCTGGCGCTGGGGGCGGAGGCAGCGCGTCTCAGGCCGTAGTGACCCAAGAAC
1392	Rv_Dl5_Vc1807_7	Cloning Constitutive dL5 reporter		R	TCGAACTGCAGGTCGACGGATCCCCGGAATTCACACTTAGTGATGGTGATGGTGATGCCC
105	BBC1881	Cloning constructs at Vc_1807 locus		F	TCGAACTGCAGGTCGACGGATCCCCGGAATTCACACTTAGTGATGGTGATGGTGATGCCC
106	BBC1882	Cloning constructs at Vc_1807 locus		R	TCGAACTGCAGGTCGACGGATCCCCGGAATTCACACTTAGTGATGGTGATGGTGATGCCC

Supplementary Table 5. Genomes used for core-gene species phylogeny.

Species/strain	Genome accession (RefSeq)
Aliivibrio fischeri ES114	GCF_000011805
Vibrio campbellii ATCC BAA-1116	GCF_000017705
Vibrio anguillarum M3	GCF_000462975
Vibrio vulnificus	GCF_001433435
Vibrio natriegens NBRC 15636	GCF_001456255
Vibrio natriegens	GCF_001680045
Vibrio natriegens	GCF_001680085
Vibrio parahaemolyticus	GCF_002073775
Vibrio anguillarum	GCF_002211985
Vibrio anguillarum	GCF_002310335
Vibrio cholerae	GCF_002313005
Escherichia coli	GCF_003018335
Vibrio campbellii	GCF_003312585
Pseudomonas aeruginosa	GCF_003369735
Vibrio campbellii	GCF_003691485
Pseudomonas aeruginosa	GCF_006971785
Escherichia coli	GCF_008926145
Vibrio cholerae	GCF_009763665
Vibrio vulnificus	GCF_009764095
Escherichia coli O55:H7	GCF_013343615
Escherichia coli O145	GCF_014607575
Vibrio parahaemolyticus	GCF_019321785
Vibrio cholerae	GCF_019704255
Vibrio parahaemolyticus	GCF_021730045
Pseudomonas aeruginosa	GCF_022638175
Aliivibrio fischeri ATCC 7744	GCF_023983475
Pseudomonas aeruginosa	GCF_025908315
Escherichia coli O2:K2:H1	GCF_025946525
Pseudomonas aeruginosa	GCF_027570555
Vibrio vulnificus	GCF_029990625
