# Supplementary material for: Cell-lysis sensing drives biofilm formation in Vibrio cholerae
Source: Nat Commun. 2024 Mar 6;15:2018. doi: 10.1038/s41467-024-46399-1 (PMC10914755; doi:10.1038/s41467-024-46399-1)
Supplement: Supplementary file 3 — Description of Additional Supplementary Files [file 41467_2024_46399_MOESM3_ESM.pdf]

Description of Additional Supplementary Files:

Supplementary Movie 1: Cross-sectional view of the *V. cholerae* biofilm response to phage-mediated lysis acquired by spinning-disc confocal microscopy. Live-cell signal from the constitutively expressed dL5-MG-2p complex is false-colored in magenta and SYTOX dead-cell signal is green.

Supplementary Movie 2: Brightfield movies of the responses to lysate for norspermidine pathway mutants.

Supplementary Movie 3: Brightfield movies of the responses to phage for norspermidine pathway mutants.

Supplementary Movie 4: Brightfield movies of the responses to lysates derived from *Vibrios* and non-*Vibrios* for wildtype *V. cholerae*.

Supplementary Movie 5: Brightfield movies of the responses of *V. anguillarum*, *V. parahaemolyticus*, and *V. vulnificus* to norspermidine and their own lysates.
